# Prevalence of bovine tuberculosis in slaughtered cattle and factors associated with risk of disease transmission among cattle handlers at Oko-Oba Abattoir, Lagos, Nigeria

**DOI:** 10.14202/vetworld.2020.1725-1731

**Published:** 2020-08-29

**Authors:** Musiliu Abiodun Agbalaya, Olayinka Olabisi Ishola, Hezekiah Kehinde Adesokan, Olufunmilayo Ibitola Fawole

**Affiliations:** 1 Nigeria Field Epidemiology and Laboratory Training Program, Nigeria; 2 Department of Veterinary Public Health and Preventive Medicine, Faculty of Veterinary Medicine, University of Ibadan, Nigeria; 3 Department of Epidemiology and Medical Statistics, Faculty of Public Health, College of Medicine, University of Ibadan, Nigeria

**Keywords:** abattoir, bovine tuberculosis, cattle handlers, prevalence, public health

## Abstract

**Background and Aim:**

Bovine tuberculosis (bTB) is a zoonotic disease of major public health importance, especially in many developing countries, including Nigeria, where control measures are largely not applied, and the risks of human infection are high. This study was aimed at determining the current prevalence of bTB in slaughtered cattle and identifying factors associated with the risk of disease transmission among cattle handlers toward making informed control measures to limit human-animal interface disease transmission.

**Materials and Methods:**

Serum samples at slaughter and lesions suggestive of bTB collected during postmortem examination of 187 slaughtered cattle at the Oko–Oba Abattoir, Agege, Lagos State, Nigeria, were subjected to lateral flow and Ziehl–Neelsen (ZN) techniques, respectively. Furthermore, a structured questionnaire was interviewer-administered to 156 cattle handlers to investigate associated exposure factors to bTB infection. Data were analyzed using bivariate and multivariate logistic regression.

**Results:**

The prevalence of bTB in cattle was 25.7% and 7.0% by lateral flow technique and ZN, respectively. The seropositivity was highest in cattle with poor body condition (50.0%), then with good (36.4%) and fair (25.0%) body conditions. The questionnaire survey revealed that being in livestock handling business for >6 years (p=0.001), not knowing the mode of transmission (p=0.02) and ignoring TB lesions at slaughter (p=0.02) were exposure factors associated with increased risk of bTB infection among the cattle handlers. Further, multivariate analysis showed that those who spent more than 6 years in livestock handling were about 4 times (Adjusted odds ratio [AOR]=3.5; 95% confidence interval [CI]=1.1-7.6, p=0.01) more likely to be exposed to bTB infection than those with lesser years. Again, respondents who called the attention of meat inspectors on seeing lesions in animals were about 4 times less likely to be exposed to bTB infection than those who ignored it (AOR=0.3; CI=0.1-0.8, p=0.01).

**Conclusion:**

This study has reiterated the endemicity of bTB in cattle population in Nigeria, with the prevalence of 25.7% and 7.0% of bTB by lateral flow and ZN techniques, respectively. This portends potential risk for disease transmission at the human-animal interface, particularly at the abattoir setting. The study also identified important knowledge and practice gaps which would enable informed, all-inclusive, and well-directed programs for effective control of the disease in both human and cattle populations.

## Introduction

Bovine tuberculosis (bTB) is a zoonotic disease that causes respiratory disorders in both cattle and humans. Active animal tuberculosis (TB) outbreaks represent possible sources of infection to both animal and human populations [1,2]. The proportion of human cases of *Mycobacterium bovis* found almost exclusively in low-income countries might be up to 10% of the global human TB burden [3,4]. bTB is classified as a list B transmissible disease of public health importance and of concern to the international trade of animals and animal products because it is of socio-economic value [5]. Worldwide, there is increasing contact between humans and animals due to increasing human population density and growth, especially in poor developing countries. In these countries, livestock production offers important socio-economic, cultural, and religious pathways out of poverty. bTB is endemic and zoonotic as *M*. *bovis* is a serious public health threat in most African countries. Geographically, the burden of TB is highest in Asia and the Africa regions, with Africa accounting for about 25% of global TB incident cases [6]. Nigeria has a population of over 190 million people and is ranked fourth among the world’s 22 countries with a high TB burden [7]. bTB is endemic in the country with the previous reports showing varying prevalence in cattle and humans [8-11]. The disease is mostly detected at slaughter during meat inspection and occasionally during sporadic screening for the disease. People who attend to, and in close contact with cattle such as herdsmen, butchers, and veterinarians, as well as the general public that consume fresh milk or infected meat are exposed to bTB [12]. This situation is worsened by the prevalence of other health and social problems such as HIV/acquired immune deficiency syndrome, smoking, drug addiction and alcoholism, and among livestock workers. Unfortunately, many of these occupationally exposed individuals are unaware of their health status, leading to considerable delays in seeking health care. These problems are further complicated by the lack of bTB control measures in Nigeria, facilitating the transmission of zoonotic infection [13].

Lagos State ranks first among the states with the highest burden of human TB in Nigeria. Besides, it accommodates one of the major slaughter facilities in the country, which supplies meat to the teeming population in the state and the neighboring environments. Considering the state as a confluent point whereby people from different walks of life seek to congregate, drawing up control programs against zoonotic TB will not only be locally beneficial but also a global necessity. Central to such an informed control program is adequate knowledge of the current status of the disease in cattle and associated exposure factors among the occupationally exposed individuals in the state.

This study was, therefore, aimed at determining the prevalence of bTB in slaughtered cattle and factors associated with risk of disease transmission among cattle handlers at the Government Abattoir, Oko-Oba, Agege, Lagos State, Nigeria.

## Materials and Methods

### Ethical approval and informed consent

Approval for this study was obtained from the Lagos State Ministry of Agriculture, and permission was also obtained from the Management of Lagos State Government Abattoir at Oko-Oba. Informed consent was obtained from each respondent before the questionnaire administration and sample collection. The confidentiality of information obtained was assured and maintained.

### Study period and site

The study was conducted at the Lagos State Government Abattoir, Oko-oba, Agege, Southwest Nigeria, between January and May 2017. The state is the economic nerve center and arguably the most populous state in the country. It accommodates one of the biggest abattoirs in the country, receives cattle from Northern Nigeria as well as from neighboring countries including Niger, Chad, Burkina Faso, Mali, and Cameroon and supplies meat to the teeming population of Lagos State. Besides, the abattoir is the biggest meat processing facility in Lagos State and serves as a source of employment for many people who derive their daily livelihood through the operations and activities that take place there. There were at least up to 120 people who were directly involved in cattle processing-related activities at the abattoir per time, aside from those who engaged in other activities such as trading.

### Study design and sample size determination

This cross-sectional study was carried out on slaughtered cattle and cattle handlers at Oko-Oba Abattoir, Agege. The minimum sample size required for slaughtered cattle for this study was calculated based on an earlier prevalence of bTB of 12.5% [14], giving a value of 187, including 10% non-response rate. On the other hand, the sample size for cattle handlers was calculated following the 28.1% previous knowledge level of bTB among cattle handlers [15], giving a minimum sample size of 133 respondents**.**

### Sampling procedure

The study was conducted following several visits to the abattoir over a period of 5 months, spanning January to May 2017. During each visit, slaughter cattle were selected using a systematic random sampling technique, selecting one of every twenty animals for collection of blood samples and detailed postmortem examination. Up to 10 mL whole blood was collected into sterile, well-labeled vacutainer tubes following the slaughter of each of the selected animals. Thereafter, on the evisceration of the selected animals, detailed postmortem examination was conducted to inspect for the presence of lesions suggestive of TB, which was collected into sterile and appropriately labeled sample polythene. Visceral organs and lymph nodes were inspected through careful visual, palpation, and incision procedures for nodules and granulomatous lesions. Data on the cattle sampled, including age (adult or young), sex (male or female), and body conditions (good, fair, or poor) were documented. The samples were transported on ice in a cooler to the NIMR Laboratory and appropriately stored until assayed. The lesions were kept at freezing temperatures of −20°C, while the blood samples were kept at freezing temperature for not more than 3 days before processing.

### Questionnaire survey

The human population included cattle handlers at the abattoir as at the time of study who were 18 years and above and had spent at least up to 6 months in the business. The purpose of the study was explained to the participants, and they were told that participation was voluntary without any attached penalty for refusal to participate. Respondents were selected using a stratified sampling technique. Sampling frames for the different strata were prepared from abattoir workers’ register, and individuals were selected by simple random sampling using balloting. Informed consent was obtained from study participants, and confidentiality was assured. A pre-tested questionnaire was interviewer-administered to the respondents by well-trained personnel to elicit information on possible exposure factors to bTB infection. The structured questionnaire included sections on data on demographic variables – age, sex, marital status, educational qualification duration of work at abattoir, and the number of people living in residence; knowledge of the disease (mode of transmission of bTB); exposure factors (keeping animals in-house, eating, and drinking while working, drinking unboiled fresh raw meat, and consumption of meat with the lesion, medical history including history of persistent cough and duration of cough, family history of TB treatment and BCG); and preventive practices (history of previous training and willingness for re-training).

### Laboratory processing

#### Ziehl Neelsen (ZN) staining

The lesions were homogenized and light smears of each of the homogenized samples were made on clean, grease-free, properly labeled dry slides. Each smear was approximately 20 mm by 10 mm, corresponding to about 100 oil immersion fields. The smears were air-dried and then heat-fixed over bunsen flame. The slides were stained using the ZN technique. The slides with fixed smears were arranged on a staining rack over a sink. Freshly prepared carbol fuchsin was poured over the slides until the smears were completely covered, and the slides were then gently heated from below with a bunsen flame until steam rose and then allowed to stay for 5 min. The stained slides were flushed with water under the running tap, and excess water on the slide drained by tilting the slides. Again, the slides were placed on the rack and the decolorizer (acid-alcohol) was poured over the slides to cover the smears and allowed to act for 2 min. The slides were thereafter washed under the running tap. The counter-stain (methylene-blue) was poured on the slides and left to stay for 1 min before washing with water under the running tap. After this, the slides were drained, arranged vertically on a slide rack and allowed to dry naturally. They were then examined under the oil-immersion objective of a binocular microscope for the presence of acid-fast bacilli, which appeared brick red against a blue background. The ability of the samples holding on to the first dye even after the acid wash gives a positive result [16].

### Lateral flow technique (one step antigen bTB antibody rapid test)

The sera obtained from the collected blood samples were processed for the detection of anti-bTB Ab against the M. bovis MPB70 antigen using the rapid lateral-flow test (Antigen bTB Ab, BioNote Inc., Republic of Korea), as described by the manufacturer. Such chromatographic immunoassays employ unique cocktails of selected *M. bovis* antigens as both qualitative captures and detectors of specific antibodies against *M. bovis* in plasma, serum, and whole blood [17,18]. MPB83, ESAT-6, 14-kDa protein, CFP-10, MPB70, MPT63, MPT51, MPT32, MPB59, MPB64, Acr1, PstS-1, *M. bovis* purified protein derivatives, ESAT-6/CFP10 fusion protein, 16-kDa alpha-crystallin/MPB83 fusion protein, and *M. bovis* culture filtrate have been identified as the common seroreactive antigens in bTB [17,19]. The bound antibodies are visualized with the naked eye as the color band at the test device within some minutes of application [17,18]. The immunochromatographic assay using recombinant MPB70 antigen as capture and detector in a direct sandwich method detected antibodies (immunoglobulin M and immunoglobulin G) against *M. bovis*. The procedure followed the guidelines described by the manufacturer. Briefly, the test kit was removed from the foil pouch and placed on a flat dry surface. With a capillary tube, one drop (10 μL) of serum was added to the sample hole marked “S” on the test device. Three drops of buffer were thereafter dispensed into the hole. A purple band in the result window of the kit would be seen in 20 min. A color band appearing in the left section of the result window showed that the test was working properly. This band was the control line (C), while the right section of the result window indicated the test results. Another color band which appeared in the right section of the result window was the test line (T).

### Statistical analysis

The analysis of data was performed using Epi info 7 version 7.2 statistical software (Centre for Disease Control and Prevention, USA). Univariate analysis was carried out to summarize the data in person, place, and time using frequencies, proportions, and mean. Bivariate analysis was performed to determine the relationship between bTB and exposure factors. In addition, multivariate analysis (logistic regression) was carried out to determine significant exposure factors for bTB infection among cattle handlers in the study area.

## Results

### Animal sampling

One hundred and eighty-seven cattle of the total 2,500 cattle slaughtered during the study period were sampled, representing 7.5% of the total slaughtered; 66.9% of which were female. Of these 187 cattle sampled and examined, 48 (25.7%) were positive for *M. bovis* antigen using the lateral flow technique. The prevalence was higher among the females (30.4%, 38/125) than the males (16.1%, 10/62). In addition, the prevalence was least in cattle with fair body condition (25%, 8/32), followed by 36.4% (36/99) with good body condition and highest in cattle with poor body condition (50.0%, 4/8) (Table-1). Again, 13 (7.0%) of the cattle examined which were all females had visible lesions suggestive of bTB and were all (100%) positive for acid-fast bacilli indicative of bTB by ZN staining.

**Table-1:** Body condition and seropositivity of cattle slaughtered at the Oko-Oba abattoir Agege Lagos, South West, Nigeria, May, 2017.

Body Condition of Cattle	Seropositive	Confidence interval 95%	Seronegative	Confidence interval 95%	Percentage
Fair	8	(7.2-10.4)	32	(22.5-45.5)	25.0
Good	36	(23.3-56.3)	99	(70.4-84.1)	36.4
Poor	4	(1.4-7.4)	8	(6.5-15.3)	50.0

### Questionnaire survey

#### Socio-demographic characteristics of respondents

Of the 156 cattle handlers surveyed, 121 (77.6%) were males. The mean age of the cattle handlers was approximately 26.4 years, and 69.9% were married. Out of the 156 cattle handlers, 65 (41.7%) had primary education while 67 (42.9%) had secondary education and 11 (7.1%) had tertiary education, whereas 13 (8.3%) had other forms of education such as Quranic education (Table-2).

**Table-2:** Demographic characteristics of cattle handlers at Oko-Oba Agege Abattoir in Lagos, South West Nigeria, May, 2017.

Characteristic	Female (n=35)	Male (n=121)	Total (%)
Age (Mean±standard deviation)	24.5±6.0	32.5±7.2	
Marital Status			
Single	12 (34.3)	30 (24.8)	42 (26.9)
Married	18 (51.4)	91 (75.2)	109 (69.9)
Widowed	5 (14.3)	0 (0)	5 (3.2)
Education			
Primary	25 (71.4)	40 (33.1)	65 (41.7)
Secondary	7 (20)	60 (49.6)	67 (42.9)
Tertiary	3 (8.6)	8 (6.6)	11 (7.1)
Others (Quran)	0 (0)	13 (10.7)	13 (8.3)
Work duration (years)			
1-2	20 (57.1)	23 (19.0)	43 (27.6)
3-5	5 (14.3%)	38 (31.4%)	43 (27.6)
>6	10 (28.6%)	60 (49.6%)	70 (44.9)

### Factors associated with risk of bTB transmission among cattle handlers in Oko-Oba Agege Abattoir, Lagos State, Southwest Nigeria, May 2017

The results of the bivariate analysis indicate that respondents who had spent more than 6 years in livestock handling were associated with 3.1 times increased risk of exposure to bTB infection (Adjusted odds ratio [AOR]=3.1; 95% confidence interval [CI]=1.3-6.0, p=0.001) compared to those with <6 years of work experience. Respondents that did not know the mode of bTB transmission were 2.2 times more at risk of exposure than those with requisite knowledge (AOR=2.2; 95% CI=1.4-5.5, p=0.02). Sleeping in animal shed was associated with increased risk of bTB compared to those that did not practice this (Table-3).

**Table-3:** Factors associated with risk of bovine tuberculosis transmission among cattle handlers in Oko-Oba Agege abattoir in Lagos, South West, Nigeria, May, 2017.

Exposure variable	Odds ratio	95% confidence interval	p-value
Length of work at abattoir: >6/<6 years	3.1	(1.3-6.0)	0.001*
Do you know mode of transmission: Yes/No	2.2	(1.4-5.5)	0.02*
Do you sleep in animal shed: Yes/No	1.6	(0.6-4.2)	0.2
Potential risk associated with your job: Yes/No	2.0	(1.4-4.0)	0.02*
Do you eat or drink at place of work: Yes/No	0.8	(0.6-2.0)	0.2
Do you drink fresh milk: Yes/No	0.3	(0.1-0.9)	0.006*
Do you boil your milk before drinking: Yes/No	0.3	(0.2-0.9)	0.02*
Do you eat raw meat: Yes/No	1.4	(0.8-4.6)	0.7
Ever seen BTB in your slaughtered animals: Yes/No	0.2	(0.1-0.5)	0.0001*
What do you do when you see BTB lesion: Call meat inspector/ignore	2.6	(1.6-5.2)	0.02*
Do you consume meat with BTB lesions: Yes/No	2.6	(1.6-5.2)	0.02*
Have or had you ever had persistent cough: Yes/No	1.4	(0.4-6.0)	0.4
Are you vaccinated with BCG: Yes/No	0.5	(0.2-2.0)	0.3
Do you receive any training on BTB: Yes/No	0.6	(0.4-2.4)	0.4

* Values significant at≤0.05; “Yes” as reference

Besides, those that did not know the potential occupational risk associated with their cattle handling were 2.0 times more at risk than those who knew. Those who engaged in eating or drinking while handling animals were 0.8 times associated with decreased risk of exposure compared to those who did not engage in such an act. Respondents who practice boiling of milk before drinking were 0.3 times less likely to be exposed to bTB infection than those who did not boil milk before drinking.

Moreover, eating raw meat increased the risk of exposure to bTB up to 1.4 times than not eating. Those that ignore lesion, as well as those who consumed it, were 2.6 times each associated with increased risk of exposure than those who called the attention of the meat inspector and those who do not consume meat with the lesion, respectively. History of having persistent cough increases the risk of exposure 1.4 times compared to those that did not have such history.

Bivariate analysis showed that being in livestock handling for >6 years (p=0.001), not knowing the mode of transmission (p=0.02), not knowing the potential occupational risk associated with handling animals (p=0.02), not boiling fresh milk before drinking (p=0.006), and ignoring TB lesions at slaughter (p=0.02) were significant factors associated with increased risk of bTB infection among the cattle handlers.

### Multivariate analysis of factors associated with the risk for bTB transmission among cattle handlers

Multivariate analysis showed that those who spent more than 6 years in livestock handling were about 4 times (AOR=3.5; 95% CI=1.2-7.6, p=0.01) more likely to be exposed to bTB infection than those who had spent lesser years. Again, respondents who call the attention of meat inspectors on seeing lesions in animals were about 3 times less likely to be exposed to bTB infection than those who ignore it (AOR=0.3; CI=0.1-0.8, p=0.01) (Table-4).

**Table-4:** Multivariate analysis of factors associated with the risk for bovine tuberculosis transmission among cattle handlers in Oko-Oba, Agege Abattoir in Lagos, South West, Nigeria, May, 2017.

Variable	Odd ratio	95% Confidence interval	p-value
Length of work	3.50	(1.16-7.58)	0.0104*
Transmission	1.71	(0.86-3.64)	0.4751
Risk to job	1.57	(0.58-4.17)	0.3865
Drinking fresh milk	0.56	(0.17-1.33)	0.0758
Raw meat	0.81	(0.34-2.18)	0.6758
Lesion in animal	0.30	(0.12-0.76)	0.0103*
Action carried out on lesion	1.30	(0.51-3.33)	0.5851
Consume lesion	1.83	(0.75-4.45)	0.1885

* Values that remained significant in the unconditional logistic regression model

## Discussion

The present study determined current prevalence of bTB in slaughtered cattle and investigated factors associated with the risk of bTB infection among cattle handlers at the Government Abattoir, Oko-Oba, Agege, Lagos State. Our findings reiterate the endemicity of bTB in cattle population as well as the presence of factors that could facilitate disease transmission at the human-animal interface in Lagos State, Nigeria. Knowledge of the current status of a disease of public health importance as bTB given its zoonotic nature as well as factors that could enhance its zoonotic transmission among the occupationally exposed individuals remains a critical consideration for informed control measures against the disease, particularly in sub-Saharan African countries where the burden of TB is high. This information is vital to achieving the WHO’s 2030 End TB Strategy, which seeks to end the global TB epidemic by 2030. More importantly, the recently launched Road Map for Zoonotic TB by WHO/OIE/FAO/IUATLD and supported by the Stop TB Partnership’s Global Plan to End TB 2016-2020 –The Paradigm Shift, identifies people at risk of zoonotic TB as a neglected population deserving greater attention WHO/OIE/FAO/IUATLD [20].

The study revealed a 25.7% prevalence of bTB by serum analysis using the lateral flow technique. This prevalence is higher than the reports of some other studies from other parts of the country. For instance, the present finding is higher than 12.5% reported in Kaduna State, North Nigeria, using the lateral flow technique [21]. It is also higher than 18.33% prevalence reported in cattle in Gombe State, North Nigeria [22]. Besides, it is similarly higher than the value reported from Ethiopia [23], which indicated a prevalence of 15.53% using lateral flow. However, our finding is lower than 37.17% reported in cattle in the highlands of Cameroon [24]. This difference may be due to relatively higher sample size and study location of these workers since cattle in Nigeria have the history of also being imported from neighboring African countries, including Cameroon.

Again, the present study revealed a bTB prevalence of 2.8% by ZN staining of gross lesions suggestive of TB among the slaughtered cattle in the study area. This prevalence is comparable to 2.8% reported by Igbokwe *et al*. [25], but higher than 0.6% reported by Cadmus *et al*. [14]. It is, however, lower than 29.16% reported in slaughtered cattle in Bauchi State, Nigeria [26]. The difference may be due to the fact that the detection of bTB lesions, in particular, depends on the diligence and thoroughness of the inspector conducting the examination [27]. Other factors that may influence the detection of tuberculous lesions in abattoirs might include underlying parasitic infections [28] and other irregularities of abattoir meat inspection [29]. Hence, cases of missed bTB lesions are a possibility. However, the findings of 2.8% bTB suspected lesions and subsequent confirmation by ZN technique among the slaughtered cattle examined in the study area underscores the importance of postmortem meat inspection as a key tool for detecting and reducing some zoonotic diseases of public health importance like bTB. This is in agreement with a report [30], which opined that postmortem examination still remains the immediate diagnostic tool to be used in endemic slaughterhouses and abattoirs in the states of Nigeria.

Further, a markedly higher prevalence was recorded using lateral flow technique when compared with ZN staining technique. Notwithstanding, the study indicated that using anti-bovine TB Ab assays as an ancillary diagnostic test to postmortem meat inspection or ZN staining technique in cattle could significantly improve the diagnosis of bTB cases. This becomes more important in such a situation where detailed meat inspection is often difficult to conduct like in most Nigerian abattoirs considering such factors as uncooperative attitudes of the butches, sharp practices by the butchers whereby they tend to hide infected organs, as well as the inadequate number of meat inspectors compared to the volume of daily slaughter [14]. The use of lateral flow technique also allows for routine screening of cattle herds for bTB without having to slaughter; thus, enabling the culling of positive reactors to limit disease progression and transmission.

The observation of more female cattle being infected than the males in this study is consistent with the previous reports [31,32], which indicated the females having more lesions than the males. With the cow having a higher prevalence, there is an implication of the infection being shed for a long time, since female cattle are kept for a longer time in the herds for the purpose of reproduction. Hence, this potentiates spreading of the disease to other animals through oral and nasal routes and to the handlers and the public through the consumption of unpasteurized milk and close proximity to infected animals. The seroprevalence based on the body condition scores of the cattle was 25%, 36.4%, and 50% among the cattle with fair, good, and poor body conditions, respectively. The observed differences were not significant, which concurs with an earlier study [32], where the body condition scoring of the nearly 3000 animals suggested no significant differences between tuberculin reactors and non-reactors.

Our findings further reveal longer duration in cattle handling (>6 years) and ignoring TB lesions at slaughter were significant factors associated with possible bTB infection among the cattle handlers in the study area. Expectedly, longer contact time with animals increases the chances of being infected with animal pathogens. This is supported by a previous report which showed that cattle handlers with longer duration in cattle handling had higher chances of being infected with bTB than those who had spent lesser time [33]. However, livestock workers, in general, lack adequate knowledge of bTB prevention [33]. Worse still, practices that enhance bTB transmission, such as consumption of unpasteurized milk and processing of infected carcasses, are prevalent among the occupationally exposed individuals [33]. Besides, the study identified important knowledge and practice gaps, including not knowing the mode of transmission of bTB, not knowing the potential occupational risk associated with handling animals, not boiling fresh milk before drinking and ignoring TB lesions at slaughter, among others. These findings are useful tips for drawing all-inclusive informed control programs against bTB transmission to humans.

The study, however, had some limitations. One, cultural isolation and molecular techniques were not conducted on the lesions collected; this would have provided more insights into the species of *Mycobacterium tuberculosis* complex responsible for the bTB in cattle in the study area. Two, sputum samples of the cattle handlers were not collected, as this would have enabled us to establish risk factors rather than associated factors for bTB transmission to the cattle handlers; thus, giving more credence to zoonotic risks associated with handling bTB infected cattle among the cattle handlers in the abattoir.

## Conclusion

This study has reiterated the endemicity of bTB in cattle as well as prevalence of factors that could enhance its transmission to humans at the human-animal interface, particularly at the abattoir setting. The current prevalence of 25.7% and 2.8% of bTB by lateral flow and ZN techniques, respectively, portends potential risk to both the cattle handlers and the general public. The study also revealed important knowledge and practice gaps which would enable informed, all-inclusive, well-directed programs for effective control against the disease in both human and cattle populations. There is a need to strengthen existing control programs through enforcement of routine surveillance for bTB in cattle as well as among the occupationally exposed individuals.

## Authors’ Contributions

OOI and MAA conceived and designed the study, MAA collected and analyzed samples under the supervision of OOI and OIF. MAA, OOI, HKA, and OIF did the statistical analysis. MAA and HKA drafted the manuscript, OOI, HKA, and OIF revised the manuscript and all authors read and approved the final manuscript.

## Competing Interests

The authors declare that they have no competing interests.

## Publisher’s Note

Veterinary World remains neutral with regard to jurisdictional claims in published institutional affiliation.
